# Mechanism of the analgesic effect of electroacupuncture on bone cancer pain through the neuro-immune system: progress based on animal experiments

**DOI:** 10.3389/fneur.2025.1693978

**Published:** 2025-12-10

**Authors:** Pengfei Qi, Mingyuan Zhou, Wenqing Han, Hongxiang Li, Lu Min, Jiaxin Li, Zhongren Sun, Hongna Yin

**Affiliations:** 1 Heilongjiang University of Chinese Medicine, Harbin, China; 2 Second Hospital of Heilongjiang University of Traditional Chinese Medicine, Harbin, China

**Keywords:** electroacupuncture, bone cancer pain, morphine tolerance, mechanism, neuroscience, immunity, animal model

## Abstract

This article summarizes the mechanism of the analgesic effect of electroacupuncture (EA) on BCP by analyzing the progress of animal experimental research on the treatment of bone cancer pain (BCP). As a kind of chronic specific pain that both overlaps and incompletely coincides with inflammatory pain and neuropathic pain, BCP is mostly clinically treated with opioids such as morphine as the first-line analgesic for BCP, but the long-term application is prone to a series of unavoidable side-effects, such as tolerance, dependence, as well as cognitive impairment, nausea, constipation, and nephrotoxicity. Therefore, there is an urgent need to seek safer and more effective treatment measures. As a safe, reliable and consistently efficacious analgesic, EA produces analgesic effects in inflammatory pain, neuropathic pain and BCP. EA not only attenuates the nociceptive sensitization of BCP by modulating the release of nociception-related neurotransmitters and receptors in the nervous system, but also exerts analgesic effects on BCP by modulating the expression of inflammatory factors in the immune system, inhibiting glial cell activation, and T cell proliferation. At present, EA research on the analgesic mechanism of BCP has made some progress, but there are still problems that need to be solved, such as the lack of standardization of acupoints and parameters, weak clinical validation, a single research model, and limitations in the perspective of analgesic mechanism research. It is suggested that future studies should be based on databases such as AcuEBase v1.0 to develop standardized EA acupoint combinations and frequency parameters to provide a scientific basis for EA standardization. At the same time, the sample size should be expanded and the experimental design should be improved in order to promote the transformation of animal experiments into clinical applications. In addition, it should be expanded to more types of cancer bone metastasis pain models to verify the consistency of the analgesic effect of EA in different BCP models. Future studies could also explore the multi-target synergistic analgesic effects of EA from a microbial-immune axis perspective with the help of tools such as MicrobeTCM. And optimize the treatment plan of BCP through EA combined with drug therapy. It is believed that with the progress of science and technology as well as the continuous exploration of human beings, the complex mechanism of BCP will be overcome by human beings eventually.

## 1 Introduction

Pain is a sensory and emotional experience associated with tissue damage or described in terms of such damage (1). The International Association for the Study of Pain (IASP) further defines pain as “an unpleasant sensory and emotional experience associated with actual or potential tissue damage, or a similar injury as described” and defines bone cancer pain (BCP) as the pain caused by a primary bone tumor (e.g., osteosarcoma) or metastatic bone tumor (e.g., breast cancer, prostate cancer bone metastasis). BCP is defined as chronic pain caused by primary bone tumors (e.g., osteosarcoma) or metastatic bone tumors (e.g., bone metastases from breast cancer and prostate cancer) (2, 3). As a chronic specific pain that both overlaps and does not fully coincide with inflammatory and neuropathic pain, BCP has the specificity of bone homeostatic changes dominated by increased osteoclast activity and bone destruction (4, 5). Pain serves as one of the most common symptoms in cancer patients. According to statistics (6, 7), more than 1.5 million cancer patients worldwide develop bone metastases, and about 60–84% of patients with advanced cancer develop bone pain of varying degrees. This not only has a serious impact on patients’ quality of life and psychological well-being, at the same time, adds to the global economic burden. Breast cancer, as the most common malignant tumor in the world, and bone tissue, as the most common metastatic site of breast cancer, BCP has long been a major problem in the global medical field that needs to be solved due to its high incidence rate as well as low treatment rate (8, 9).

Currently, clinical treatment of BCP is still based on the “three-step therapy” recommended by the World Health Organization (WHO), which starts with non-opioid analgesics and progresses to opioid analgesics as pain increases (10). Initially, with the pain-relieving effect of analgesic drugs, the pain of BCP patients has been effectively relieved. However, long-term use of opioid analgesics such as morphine will not only lead to morphine tolerance, resulting in “failure” of analgesics, but also addiction and dependence, as well as cognitive impairment, nausea, constipation, nephrotoxicity and a series of other unavoidable side effects (11, 12). Therefore, the search for safer and more effective analgesic regimens and the alleviation of the phenomenon of tolerance after morphine treatment is a hot topic at the forefront of the oncological pain field today.

The study shows that (13), the combination of low-level laser therapy with conventional physical therapy favors pain relief. In recent years, with the increasing research on traditional Chinese medicine, several studies in the literature have reported that electroacupuncture (EA) has a significant analgesic effect on BCP, and, moreover, EA is also effective in alleviating the BCP-morphine tolerance phenomenon (14–17). EA is a therapy that involves the insertion of millimetre needles into acupoints of the body, and by accessing an electroacupuncture device, the co-stimulatory effect of needles and electric current is utilized to prevent and treat diseases. It is now widely used in the treatment of all types of pain (18). However, acupuncture analgesia, as a complex network regulatory mechanism, not only involves the entire nervous system from the periphery to the center, but is also closely related to the immune system. Many bioactive substances are involved in the regulation of BCP analgesic effects by EA. Currently, the study of BCP mechanism is mainly based on animal experiments, mostly using one side of the tibia or femur to inject breast cancer cells to construct experimental animal models (19, 20). In view of the complex pathogenesis of BCP and the good analgesic effect of EA on BCP, this paper summarizes the animal experimental study on the mechanism of analgesic effect of EA on BCP. It is found that the analgesic effect of EA on BCP is mainly realized by affecting the release and expression of neurotransmitters, cytokines and immune cells in the nervous system and immune system. In the following, we will discuss the mechanism of the analgesic effect of EA on BCP by focusing on the nervous system, which is composed of the release of neurotransmitters and receptors related to nociception, and the immune system, which is composed of the expression of inflammatory factors, the activation of glial cells, and the proliferation of T cells, respectively. In order to provide a theoretical basis for future in-depth investigation of the analgesic mechanism of EA on BCP, we summarize as follows.

## 2 The analgesic mechanism of EA on BCP

The analgesic mechanism of EA on BCP mainly involves the nervous system which consists of the release of neurotransmitters and receptors, and the immune system which consists of the expression of inflammatory factors, the activation of glial cells, and the proliferation of T cells. The analgesic mechanism of EA on BCP is shown in Table 1 and the analgesic mechanism of EA on BCP-morphine tolerance is shown in Table 2.

**Table 1:** The analgesic mechanism of EA on BCP.

Acupoint	Frequency	Site of action	Mechanism of action	Effect	Ref.
ST36 BL60	2/100 Hz	DRG	P2X3R↓	Downregulation of P2XR expression	(29)
ST36 BL60	100/200 Hz EA	SDH	ETAR↓, PI3K↓, P-Akt↓	Downregulation of ET receptor expression	(38)
ST36 BL60	2/100 Hz	RVM, spinal cord	5-HT↓, 5-HT3AR↓	Down-regulates the expression of 5-HT and its receptor 5-HT3AR	(45)
GB30	2 Hz	Spinal cord	CGRP↓, p75↓, TrkA↓	Downregulation of neuropeptide and its receptor expression	(58)
ST36 BL60	2/100 Hz	RVM, spinal cord	MOR↑, EM-1↑	Regulation of EOP and its receptor expression	(45)
ST36 SP6	2 Hz	BLA	PPD↑	Regulation of EOP and its receptor expression	(60)
GB30	10 Hz	SDH	IL-1β↓	Regulation of inflammatory factor expression	(74)
ST36 SP6	2 Hz	BLA	TNFSF8↓	Regulation of inflammatory factor expression	(60)
GB30	2 Hz	Spinal cord	NF-κB↓, COX-2↓, IL-1β↓	Regulation of inflammatory factor expression	(58)
ST36 BL60	2/100 Hz	vlPAG	pNF-κB↓, CXCL 12↓	Inhibition of glial cell activation	(92)
ST36 BL60	2/100 Hz	Spleen	CD 3^+^↑, CD 8^+^ T↑, IL-2↑	Promote T-cell proliferation	(100)

↑ indicates an increase or upward adjustment; ↓ indicates a decrease or downward adjustment.

**Table 2:** The analgesic mechanism of EA on BCP-morphine tolerance.

Acupoint	Frequency	Site of action	Mechanism of action	Effect	Ref.
ST36 BL60	2/100 Hz	DRG	HDAC1↓, MOR↑	Upregulation of MOR expression	(61)
ST36 BL60	2/100 Hz	LC	MOR↑	Upregulation of MOR expression	(62)
ST36 BL60	2/100 Hz	LC	GRK5↑, β-arrestin2↓, PKCα↓	Inhibition of MOR desensitization	(65)
ST36 BL60	2/100 Hz	LC	Rab5↑, MOR^+^/Rab5^+^↑	Promotion of MOR endocytosis	(69)

↑ indicates an increase or upward adjustment; ↓ indicates a decrease or downward adjustment.

### 2.1 Mechanism of the analgesic effect of EA modulation of the nervous system on BCP

**Figure 1:**
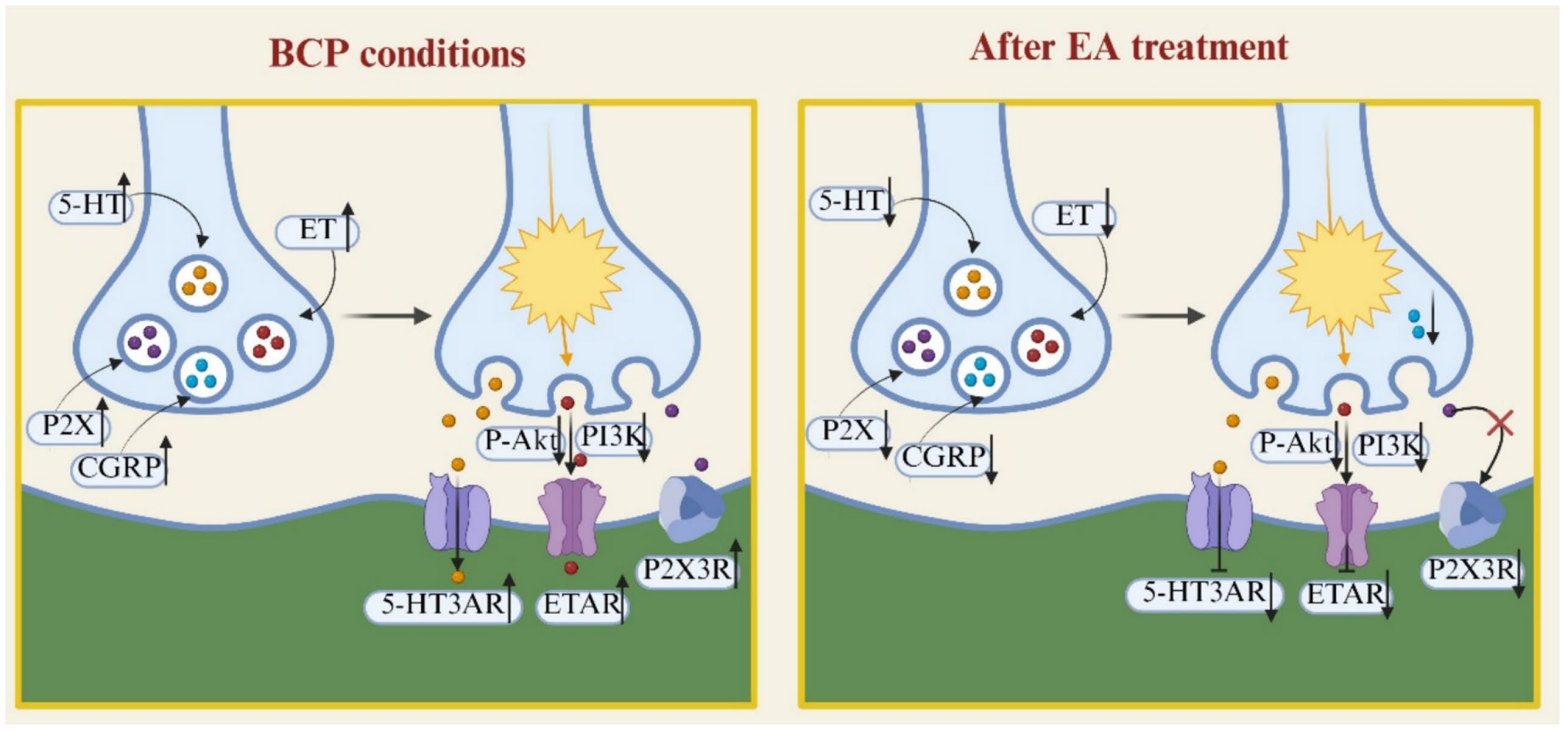
EA produces analgesic effects on BCP by down-regulating the release of neurotransmitters and receptors associated with nociception.

**Figure 2:**
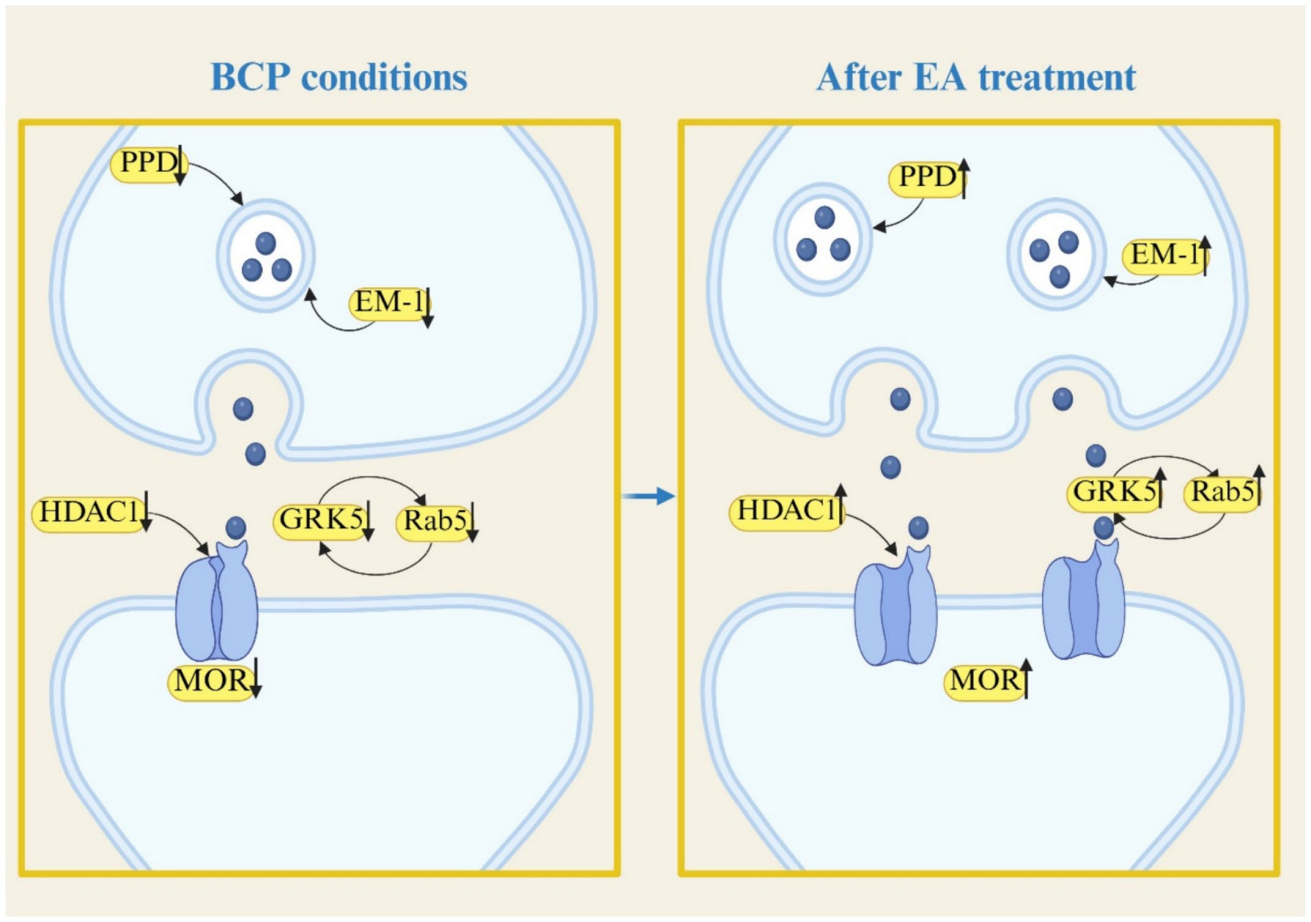
EA produces analgesic effects on BCP by upregulating the release of neurotransmitters and receptors associated with nociception.

The analgesic effect of EA on BCP through the nervous system is mainly dependent on the modulation of the release of nociception-related neurotransmitters and receptors. Specifically, EA can not only down-regulate the expression of ionotropic Purinergic P2X receptors (P2XR) and endothelin (ET) receptor to produce analgesic effects on BCP, but also attenuate the nociceptive sensitization of BCP by down-regulating the expression of 5-hydroxytryptamine (5-HT), neuropeptides and their related receptors. In addition, EA was able to produce analgesic effects on BCP by modulating the expression of Endogenous opioid peptide (EOP) and its receptor. And by inhibiting the desensitization of *μ*-opioid receptor (MOR) in the EOP receptor, it accelerated the resensitization and endocytosis of MOR, and then alleviated the phenomenon of BCP-morphine tolerance. The mechanism of the analgesic effect of EA on BCP by regulating the release of nociception-related neurotransmitters and receptors is shown in Figures 1, 2.

#### 2.1.1 Downregulation of P2XR expression

The Purinergic P2 receptors (P2R) is a cellular signaling factor that plays a crucial role in maintaining normal physiological functions in the body. This receptor is classified into two subtypes: the ionotropic P2XR and the metabotropic P2Y receptor. P2XR is mainly distributed in primary sensory neurons within the dorsal root ganglia (DRG) and other sensory ganglia, with seven subtypes including P2X1R-P2X7R (21, 22). When P2XR is activated by extracellular ATP, it regulates ion channel activity and participates in nociceptive signaling by mediating Na^+^ and Ca^2+^ inward flow as well as K^+^ outward flow.

The study shows that (23–25), functional upregulation of P2X3R, P2X7R is a potential effector mechanism leading to BCP nociceptive sensitization. For example, intrathecal injection of the P2X3R antagonist A317491 attenuates nociceptive sensitization in BCP model mice by down-regulating the expression of P2X3R in the DRG, and thus attenuates nociceptive sensitization in BCP model mice (26, 27). However, decreasing the expression of P2X7R in the hippocampus of BCP model rats produced analgesic effects (28).

2/100 Hz EA ZuSanLi (ST36) and KunLun (BL60) can reduce P2X3R overexpression and membrane transport in the DRG by downregulating the expression of P2X3R in the L4-L6 DRG and the ratio of membrane proteins to total proteins in BCP model rats, which in turn attenuates the nociceptive sensitization of BCP, and intrathecal injection of the P2X3R specific agonist *α*, *β*-meATP reversed the analgesic effect of EA (29). It is suggested that the analgesic effect of 2/100 Hz EA ST36 and BL60 on BCP model rats may be realized by decreasing the overexpression and membrane transport of P2X3R in DRG.

#### 2.1.2 Downregulation of ET receptor expression

ET is an endogenous small-molecule polypeptide analog composed of 21 amino acids widely found in the nervous system, with a total of three isoforms including ET-1, ET-2, and ET-3 (30). Among them, ET-1, a peptide hormone with multiple biological functions encoded by the EDN 1 gene, binds to two different G-protein-coupled receptors, Endothelin Receptor Type A (ETA) and Endothelin Receptor Type B (ETB), which together mediate BCP (31). ETAR preferentially binds ET-1, whereas ETBR has a similar affinity for ET-1, ET-2, and ET-3 (32).

The study shows that (33, 34), activation of ETAR stimulates sensory neurons leading to nociceptive hypersensitivity, and down-regulation of ETAR expression has an analgesic effect on BCP. For example, intraperitoneal injection of the selective ETAR antagonist BQ-123 effectively attenuated BCP nociceptive sensitization induced by ET-1 (35). Heel bone injection of BQ-123 also reduced nociceptive sensitization in BCP model mice by inhibiting C injury receptor activation (36). In addition, intrathecal injection of BQ-123 could produce analgesic effects in BCP model mice by down-regulating protein kinase B (Akt) phosphorylation in spinal cord tissues. It is suggested that down-regulation of ETAR expression could attenuate BCP nociceptive sensitization by inhibiting Akt phosphorylation (37).

100/200 Hz EA ST36 and BL60 exerted analgesic effects on BCP by down-regulating the protein expression of ETAR mRNA as well as P-PI3K and P-Akt in neurons of the spinal dorsal horn (SDH) of BCP model rats (38). It is suggested that the analgesic effect of EA on BCP is achieved by down-regulating the expression of ETAR and inhibiting the phosphorylation of the PI3K/Akt signaling pathway.

#### 2.1.3 Downregulation of 5-HT and its receptor expression

5-HT, a monoamine neurotransmitter widely found in the central nervous system (CNS), is formed by enzymatic hydroxylation of the amino acid I-tryptophan to 5-hydroxytryptophan (5-HTP), which is then decarboxylated by amino acid decarboxylase (39). 5-HT is involved in the regulation of a number of physiological functions, including mood, cognition, perception, and memory, through binding to seven receptors, including its G-protein-coupled receptor and ligand-gated cation channels (40). Among them, 5-hydroxytryptamine 3 receptor (5-HT3R), as the only ionotropic receptor, is involved in nociceptive signaling by mediating fast excitatory synaptic transmission, and its overactivation exacerbates nociceptive sensitization (41, 42).

The study shows that (43), The selective 5-HT3R antagonist ondansetron exerts analgesic effects on BCP by inhibiting SDH neuronal responses in BCP model rats. It is suggested that down-regulation of 5-HT3R expression attenuates the nociceptive sensitization of BCP. EA ST36 can produce analgesic effects by modulating the synthesis and release of 5-HT and its 5-HT3R in the CNS (44). 2/100 Hz EA ST36 and BL60 attenuated the nociceptive sensitization of BCP model rats by decreasing the mRNA expression of 5-HT and 5-HT3AR in the Rostral Ventromedial Medulla (RVM) and spinal cord tissues (45). It is suggested that the mechanism of the analgesic effect of EA on BCP may involve down-regulation of the expression of 5-HT, 5-HT3AR.

#### 2.1.4 Downregulation of the expression of neuropeptides and their receptors

Neuropeptides are peptides composed of 3–100 amino acid residues that act as messengers in the immune system to regulate neuroinflammatory responses (46). Calcitonin gene related peptide (CGRP), which is mainly localized in A and C sensory nerve fibers, as a neuropeptide composed of 37 amino acids, is the other major proinflammatory neuropeptide besides substance P. Previous studies have mostly focused on CGRP for migraine relief (47–49). The recent study shows that (50, 51), CGRP can be involved in nociceptive signaling, integration, and modulation by binding to the receptor tropomyosin receptor kinase A (TrkA), which activates injury receptors, and to the P75 neurotrophin receptor (P75NTR). Abnormally increased activity of CGRP, a key regulator in the bone microenvironment, drives bone metastasis and thus triggers BCP, whereas inhibition of CGRP activity produces analgesic effects on BCP (52, 53). Furthermore, overexpression of TrkA with P75NTR exacerbates nociceptive sensitization of BCP by promoting breast cancer cell growth, migration, and invasion, which in turn exacerbates nociceptive sensitization (54). Oral administration of the selective TrkA inhibitor ARRY-470 produces analgesic effects in BCP model mice by modulating sensory nerve fiber remodeling (55). And the analgesic effect produced by down-regulation of TrkA expression on BCP may be related to the reduction of *δ*-opioid receptor (DOR) content in the DRG (56). Down-regulation of P75NTR activity attenuates the nociceptive sensitization of BCP by decreasing the expression level of the immunomodulatory kinase mammalian target of rapamycin (mTOR) in the DRG and SDH neurons of BCP model rats (57).

2 Hz EA HuanTiao (GB30) attenuate the nociceptive sensitization of BCP by down-regulating the protein expression levels of neuropeptide-related proteins CGRP, P75NTR, and TrkA in the spinal cord tissue of BCP model rats (58). It is suggested that the mechanism of the analgesic effect of EA on BCP may be realized by inhibiting the binding of CGRP to its receptors p75 and TrkA.

#### 2.1.5 Regulation of EOP and its receptor expression

EOP is an important modulator in the central system with the ability to regulate the expression and activity of opioid receptors, which mainly consists of *β*-endorphin (β-EP), enkephalin, and dynorphin (Dyn). EOP exerts analgesic effects on BCP through its action on *μ*, *δ*, and *κ* opioid receptors (MOR, DOR, and κOR) (59). EA can attenuate the nociceptive sensitization of BCP by modulating the expression of EOP and its receptor. 2/100 Hz EA ST36 and BL60 produces analgesic effects on BCP by up-regulating the protein as well as the mRNA expression of MOR in the spinal cord tissue of the rat model of BCP and by up-regulating the content of the endogenous ligand of MOR, Endomorphin-1 (EM-1), in the spinal cord tissue and RVM (45). In addition, 2 Hz EA ST36 and SanYinJiao (SP6) also upregulate the mRNA expression level of prodynorphin (PPD) in the Basal lateral amygdala (BLA) of BCP model rats, and attenuate the nociceptive sensitization of BCP model rats by promoting the synthesis of Dyn and upregulating the expression of EOP (60).

EA also alleviate BCP-morphine tolerance by up-regulating the expression of MOR. 2/100 Hz EA ST36 and BL60 alleviate morphine tolerance in BCP rats by down-regulating the protein expression of histone deacetylase 1 (HDAC1) in DRG, which in turn up-regulated the protein expression of MOR (61). 2/100 Hz EA ST36 and BL60 also alleviate morphine tolerance in BCP rats by increasing MOR immunofluorescence-positive expression in the locus coeruleus (LC) of BCP-morphine tolerance model rats (62). The LC is enriched with a large number of MORs, and MOR desensitization and its reduced ability to resensitize is a key mechanism leading to BCP-morphine tolerance (63). Therefore, inhibiting MOR desensitization and promoting its resensitization is an effective strategy to alleviate morphine tolerance. The study shows that (64), after BCP-morphine tolerance, the binding efficiency of G Protein-Coupled Receptor Kinase 5 (GRK5) to the soluble protein *β*-arrestin is accelerated, leading to an up-regulation of protein kinase C (PKC) activity and its level of phosphorylation, enhancing MOR desensitization and exacerbate the phenomenon of tolerance. 2/100 Hz EA ST36 and BL60 alleviated the BCP-morphine tolerance phenomenon by up-regulating the protein expression of GRK5 in the LC of BCP-morphine tolerance model rats, while decreasing the protein expression of the soluble proteins *β*-arrestin2, PKCα, and their phosphorylated proteins (65). It is suggested that the inhibition of MOR desensitization by EA to alleviate the morphine tolerance phenomenon in BCP model rats may be achieved by inhibiting the binding of GRK5 to β-arrestin2, which in turn down-regulates PKCα activity and its phosphorylated protein expression.

Furthermore, multiple studies have shown that (66, 67), MOR endocytosis can inhibit opioid receptor desensitization, thereby reducing morphine tolerance and dependence. For example, MOR-targeting monoclonal antibodies can alleviate BCP-morphine tolerance by enhancing morphine-induced MOR endocytosis (68). 2/100 Hz EA at ST36 and BL60 alleviated morphine tolerance in BCP-morphine tolerance model rats by increasing the rate of MOR-positive cells expressing the endocytic protein Rab5 and the MOR^+^/Rab5^+^ positive rate in the LC (69). This suggests that EA alleviates morphine tolerance in BCP model rats by enhancing MOR expression in the LC and promoting MOR endocytosis.

### 2.2 Mechanism of the analgesic effect of EA modulation of the immune system on BCP

**Figure 3:**
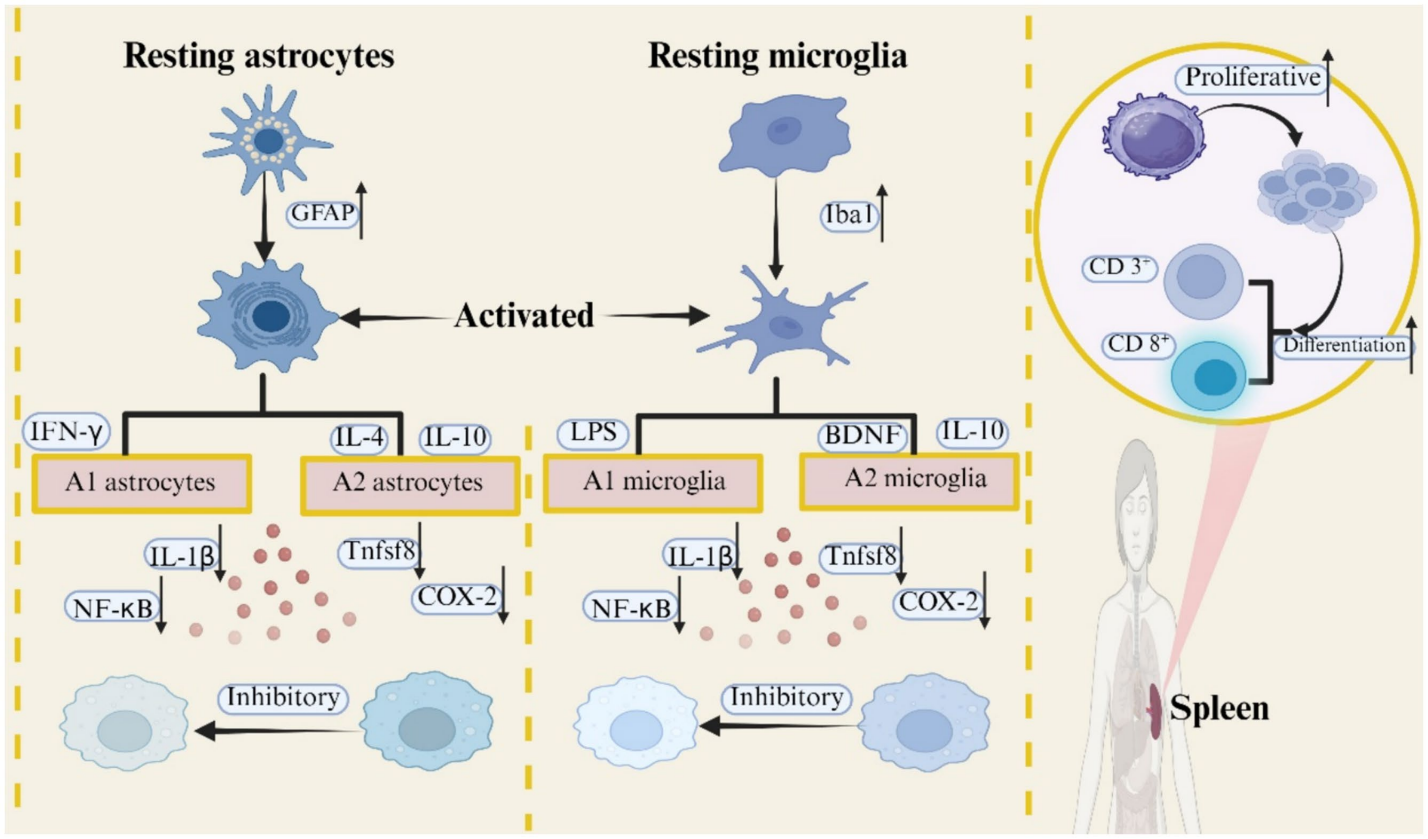
Mechanism of EA analgesia in BCP through immunomodulation.

The analgesic effect of EA on BCP through the immune system is mainly dependent on modulating the expression of inflammatory factors, inhibiting glial cell activation, and promoting T cell proliferation. Specifically, EA not only attenuates the nociceptive sensitization of BCP by down-regulating the expression of inflammatory factors such as Interleukin-1Beta (IL-1β), tumor necrosis factor-*α* (TNF-α), tumor necrosis factor superfamily 8 (TNFSF8), and nuclear factor kappa-B (NF-κB). It also produce analgesic effects on BCP’s by inhibiting the activation of microglia and astrocytes. In addition, the analgesic effect of EA on BCP is also closely related to the proliferation of T cells and their subtypes CD 4^+^ and CD 8^+^ T cells. The analgesic mechanism of EA on BCP through the immune system is shown in Figure 3.

#### 2.2.1 Regulation of inflammatory factor expression

BCP formation is closely associated with inflammatory cytokine-mediated chemical signaling (70, 71). As the major pro-inflammatory factor produced by myeloid cells, IL-1β is the most well characterized and studied member of the IL-1 family. In physiologic states, IL-1β is secreted at low levels, whereas in pathological conditions, IL-1β expression is significantly elevated (72). The study shows that (73), IL-1β was significantly elevated in the spinal cord tissue of BCP model mice, and intrathecal injection of the IL-1 receptor antagonist, Anakinra, attenuated the nociceptive sensitization of BCP by down-regulating the expression of IL-1β in spinal cord tissue. It is suggested that down-regulation of IL-1β expression could have an analgesic effect on BCP. 10 Hz EA GB30 attenuated nociceptive sensitization of BCP by down-regulating RNA expression of IL-1β in layers I-V within the SDH of BCP model rats, and EA inhibited IL-1β in deeper layers of the SDH more strongly than in superficial layers (74). It is suggested that the attenuation of nociceptive sensitization of BCP by EA may be partly dependent on the down-regulation of IL-1β expression.

In addition, macrophage-derived TNF-*α* also has strong proinflammatory activity. The progression of BCP is accompanied by a high production of TNF-*α*, and down-regulation of its expression can have an analgesic effect on BCP (75). The study shows that (76), the expression of both TNF-α and its receptor TNFR 1 was significantly elevated in the DRG of BCP model rats. Down-regulation of the expression of TNF-α and its receptor TNFR 1 attenuated nociceptive sensitization in BCP. Inhibition of the expression of TNFSF8, a member of the TNF superfamily, similarly produces an analgesic effect on BCP by downregulating the inflammatory response (77). 2 Hz EA ST36 and SP6 attenuate the nociceptive sensitization of BCP by down-regulating the level of TNFSF8 expression in BLA, and thus attenuate the nociceptive sensitization of BCP (60). It is suggested that EA may have an analgesic effect on BCP by down-regulating the expression of TNFSF8 and attenuating the inflammatory response.

NF-κB, an important transcription factor in the organism, is extensively involved in the regulation of nociceptive signaling. Inhibition of NF-κB activation attenuates nociceptive hypersensitivity in BCP (78). For example, intrathecal injection of the NF-κB inhibitor BAY11-7081 produced an analgesic effect on BCP by down-regulating the expression of NF-кB in neurons and astrocytes in the spinal cord tissue of BCP model rats and up-regulating the expression of the anti-inflammatory factor Interleukin-4 (IL-4) (79). In addition, 2-Hz EA GB30 attenuated the nociceptive sensitization of BCP by decreasing the protein expression levels of NF-κB, Cyclooxygenase-2 (COX-2), and IL-1β in the spinal cord tissues of BCP model rats, which in turn attenuated the nociceptive sensitization of BCP (58). It is suggested that the analgesic effect of EA on BCP is related to the inhibition of NF-κB activation and the down-regulation of pro-inflammatory factor expression.

#### 2.2.2 Inhibition of glial cell activation

The CNS is composed of neurons and glial cells, where glial cells mainly include microglia, astrocytes, and oligodendrocytes (80). The study shows that (81), Continued activation of microglia and astrocytes promotes the development and progression of BCP. Microglia, originating from the yolk sac, are the earliest glial cells in the CNS to respond to injury and can be involved in BCP regulation through immunomodulation. Their phenotypic polarization occurs in response to different stimuli and exerts a pro- or analgesic effect on BCP. Physiologically, M1 and M2 microglia are in a dynamic state of equilibrium. When microglia undergo activation, this equilibrium is disrupted, thereby inducing BCP (82). Stimulated by Interferon-*γ* (IFN-γ) and lipopolysaccharide (LPS), M1-type microglia enhance neuronal excitability and aggravate the nociceptive sensitization of BCP by releasing pro-inflammatory cytokines and other nociceptive mediators, whereas M2-type microglia induce the release of anti-inflammatory mediators, growth factors, and neurotrophic factors, which produce analgesic effects on BCP (83, 84). For example, intrathecal injection of the glycolysis inhibitor 2-Deoxy-D-glucose (2-DG) attenuates the nociceptive sensitization of BCP by inhibiting the polarization of M2-type microglia to M1-type, decreasing the production of the proinflammatory factors TNF-*α*, IL-1β, and IL-6, and facilitating the secretion of the anti-inflammatory factor IL-10 (85).

Astrocytes, as the most abundant and functionally complex glial cells in the mammalian CNS, are similarly involved in the genesis and development of BCP by influencing immunoregulatory mechanisms (86). Under physiological conditions, astrocytes are in a resting state and exhibit a “homeostatic” phenotype. When BCP occurs, astrocyte cytosol and protrusions are hypertrophied, elongated, and increased in number, and the expression of the intermediate filament protein glial fibrillary acidic protein (GFAP), waveform proteins, and nestin proteins is elevated, transforming them into a reactive state (87). Reactive astrocytes can be classified into two phenotypes, A1 and A2. A1-reactive astrocytes exacerbate the nociceptive sensitization of BCP by releasing pro-inflammatory cytokines such as IL-1β, IL-1*α*, and TNF-α, whereas A2-reactive astrocytes inhibit inflammatory responses by secreting anti-inflammatory factors, such as IL-10, and produce analgesic effects on BCP (71, 88). For example, microinjection of the astrocyte cytotoxin L-α-aminoadipate into the ventrolateral periaqueductal gray (vIPAG) of BCP model rats attenuates nociceptive sensitization in BCP model rats by inhibiting astrocyte activation within the vIPAG (89).

Inhibition of the sustained activation of microglia and astrocytes both have analgesic effects on BCP (90). In BCP model rats, microinjection of selective microglia fine or astrocyte inhibitors into the RVM produces analgesic effects on BCP model rats by eliminating local glial cell activation (91). In addition, 2/100 Hz EA foot Sanli and Kunlun attenuated the nociceptive sensitization in BCP model rats by inhibiting the activation of GFAP and microglia marker Iba 1 in vlPAG and downregulating the protein expression of pNF-κB and CXCL 12 in astrocytes and microglia (92). It is suggested that the analgesic effect of EA on BCP model rats may be achieved by inhibiting the activation of glial cells in vlPAG and downregulating the NF-κB/CXCL12 signaling pathway.

#### 2.2.3 Promotion of T-cell proliferation

T cells derive from bone marrow hematopoietic stem cells and their subtypes CD 4^+^ and CD 8^+^ T cells serve as key effector cells in the immune system. During the immune response, they mediate the inflammatory cascade by secreting cytokines and regulate other immune cells, which together play an immunomodulatory role (93). CD 4^+^ T cells protect mice from osteolytic lesions by inhibiting Receptor Activator of Nuclear Factor-κB Ligand (RANKL) activity (94). CD 8^+^ T cells, on the other hand, attenuate the nociceptive sensitization of BCP by producing anti-inflammatory cytokines (95). These findings suggest that the mechanism of the analgesic effect of BCP may be related to the upregulation of the expression levels of CD 4^+^ and CD 8^+^ T cells.

Interleukin-2 (IL-2) is a growth factor that drives the expansion of activated T-cell populations and has functions in promoting the differentiation of various effector T-cell populations, controlling immune responses, and maintaining self-tolerance (96). Reduced IL-2 expression on CD 8^+^ T cells can lead to decreased cytokine secretion as well as effector function by downregulating antigenic reactivity (97). Upregulation of serum IL-2 levels can attenuate nociceptive sensitization of BCP by enhancing the body’s immune response and, in turn, attenuating the nociceptive sensitization of BCP (98). Acupuncture can have an analgesic effect on BCP by elevating CD 3^+^ T-cell subsets as well as IL-2 levels and increasing the body’s immune response (99). 2/100 Hz EA ST36 and BL60 attenuated nociceptive hypersensitivity in BCP model rats by promoting the proliferation of splenic T-lymphocytes as well as the percentages of CD 3^+^ and CD 8^+^ T-cell subpopulations and up-regulating plasma levels of the T-cell growth factor IL-2 (100). It is suggested that the analgesic effect of EA on BCP may be realized by promoting the proliferation of T lymphocytes.

## 3 Discussion

BCP is one of the most common symptoms in cancer patients. In recent years, the number of BCP patients has increased rapidly with the rising incidence of cancer. Compared with the irreversible secondary damage caused by radiotherapy, chemotherapy and surgery, oral or intramuscular injection of analgesic drugs to relieve BCP is a safer, more reliable and feasible option with relatively few side effects. The WHO has proposed a “three-step approach” to address the complex mechanisms of BCP, but the long-term use of opioids (e.g., morphine) in this approach can lead to drug tolerance, which reduces the analgesic effect. To address this issue, Chinese researchers were the first to introduce EA into animal studies of BCP, aiming to evaluate its analgesic effect and whether it is accompanied by morphine tolerance. Animal experiments show that EA not only effectively reduced the nociceptive sensitization of BCP, but also improved the morphine tolerance phenomenon. These findings have promoted the in-depth study of EA in BCP.

Current research has found that EA exerts analgesic effects mainly through modulating the nervous and immune systems, and the specific mechanisms include targeting neurotransmitters, regulating the expression of inflammatory factors, and influencing glial cell activation and T cell proliferation. Although EA has made some progress in BCP research, the following issues still need to be addressed:

- Lack of standardization of EA acupoints and parameters: acupoint specificity has long been controversial in the field of acupuncture (101). Currently, the selection of acupoints for animal experimental studies of EA to alleviate BCP is dominated by acupoints such as ST36 and BL60. The stimulation frequency is mostly 2/100 Hz, and a uniform standard has not yet been formed. It is suggested that in subsequent animal experiments, the combination of acupoints and stimulation parameters should be systematically optimized with the help of databases such as AcuEBase v1.0 to provide a scientific basis for the standardization of EA (102).
- Weak clinical validation: At this stage, animal experimental studies generally suffer from small sample sizes and lack of rigor in experimental design. This limits the statistical reliability and credibility of the study conclusions and hinders the translation to high-quality randomized controlled trial (RCT). In the future, sample size should be enlarged and experimental design should be improved on the basis of standardization in order to improve the clinical translation rate and narrow the gap between preclinical discovery and clinical application.
- Single research model: Breast cancer patients are prone to bone metastasis, which further leads to BCP. Therefore, EA studies on the mechanism of analgesic effect of BCP are mostly conducted in female animal models constructed by injecting breast cancer cells into one side of tibia or femur. However, other cancers, such as prostate, liver, and lung cancers, also have bone metastasis of varying degrees, which also leads to the occurrence of BCP. The lack of experimental animal studies modeling bone metastases from other cancers in the current study has led to the question of whether there is variability in the analgesic effect of EA between BCP models induced by different cancers has not been effectively addressed. For example, the animal models selected for prostate cancer and breast cancer are affected by hormone levels, and there is a lack of relevant experiments to verify whether EA modulation of the same targets can still exert the same analgesic effect. In the future, we should expand to more types of cancer bone metastasis pain models to verify whether the analgesic effect of EA is consistent in different BCP models.
- Limited perspectives in the study of analgesic mechanisms: Evidence of whether many of the mechanisms that have been identified in inflammatory and neuropathic pain (e.g., Ca^2+^/calmodulin kinase II phosphorylation, noncoding RNA expression, dendritic spine remodeling, endoplasmic reticulum stress, glucose metabolism, etc.) also attenuate nociceptive sensitization to BCP remains to be validated by relevant experiments (103). In addition, the study of EA at the level of central brain regions is far less in-depth than that at the level of spinal cord. It is recommended to systematically explore the pain regulatory networks between brain regions and between the brain-spinal cord by combining techniques such as neuronal tracing, and to expand the multi-target synergistic mechanism of EA from the microbe-immunity axis perspective with the help of tools such as MicrobeTCM (104).

Future studies may also provide better treatment options for BCP and morphine tolerance through EA combination therapy. It is believed that with the advancement of science and technology and the continuous exploration of mankind, many outstanding problems of BCP will be solved, and the complex mechanism of BCP will be conquered by mankind.

## Glossary

Akt: Protein kinase B
BCP: Bone cancer pain
BLA: Basal lateral amygdala
β-EP: Beta-endorphin
CNS: Central nervous system
CGRP: Calcitonin gene related peptide
COX-2: Cyclooxygenase-2
Dyn: Dynorphin
DRG: Dorsal root ganglia
DOR: δ-opioid receptor
EA: Electroacupuncture
EOP: Endogenous opioid peptide
EM-1: Endomorphin-1
ET: Endothelin
ETA: Endothelin Receptor Type A
ETB: Endothelin Receptor Type B
GFAP: Glial Fibrillary Acidic Protein
GRK5: G Protein-Coupled Receptor Kinase 5
HDAC1: Histone deacetylase 1
IASP: International Association for the Study of Pain
IL-1β: Interleukin-1Beta
IL-4: Interleukin-4
IFN-γ: Interferon-γ
IL-2: Interleukin-2
LC: Locus Coeruleus
LPS: Lipopolysaccharide
MOR: μ-opioid receptor
mTOR: Mammalian target of rapamycin
NF-κB: Nuclear factor kappa-B
P2XR: Purinergic P2X receptors
P2R: Purinergic P2 receptors
P75NTR: P75 neurotrophin receptor
PPD: prodynorphin
PKC: Protein kinase C
RVM: Rostral Ventromedial Medulla
RANKL: Receptor Activator of Nuclear Factor-κB Ligand
RCT: Randomized Controlled Trial
SDH: Spinal dorsal horn
TrkA: Tropomyosin receptor kinase A
TNF-α: Tumor necrosis factor-α
TNFSF8: Tumor necrosis factor superfamily 8
vIPAG: Ventrolateral periaqueductal gray
WHO: World Health Organization
κOR: κ-opioid receptor
5-HT: 5-hydroxytryptamine
5-HTP: 5-hydroxytryptophan
5-HT3R: 5-hydroxytryptamine 3 receptor
2-DG: 2-Deoxy-D-arabino-hexose
Akt: Protein kinase B
BCP: Bone cancer pain
BLA: Basal lateral amygdala
β-EP: Beta-endorphin
CNS: Central nervous system
CGRP: Calcitonin gene related peptide
COX-2: Cyclooxygenase-2
Dyn: Dynorphin
DRG: Dorsal root ganglia
DOR: δ-opioid receptor
EA: Electroacupuncture
EOP: Endogenous opioid peptide
EM-1: Endomorphin-1
ET: Endothelin
ETA: Endothelin Receptor Type A
ETB: Endothelin Receptor Type B
GFAP: Glial Fibrillary Acidic Protein
GRK5: G Protein-Coupled Receptor Kinase 5
HDAC1: Histone deacetylase 1
IASP: International Association for the Study of Pain
IL-1β: Interleukin-1Beta
IL-4: Interleukin-4
IFN-γ: Interferon-γ
IL-2: Interleukin-2
LC: Locus Coeruleus
LPS: Lipopolysaccharide
MOR: μ-opioid receptor
mTOR: Mammalian target of rapamycin
NF-κB: Nuclear factor kappa-B
P2XR: Purinergic P2X receptors
P2R: Purinergic P2 receptors
P75NTR: P75 neurotrophin receptor
PPD: prodynorphin
PKC: Protein kinase C
RVM: Rostral Ventromedial Medulla
RANKL: Receptor Activator of Nuclear Factor-κB Ligand
RCT: Randomized Controlled Trial
SDH: Spinal dorsal horn
TrkA: Tropomyosin receptor kinase A
TNF-α: Tumor necrosis factor-*α*
TNFSF8: Tumor necrosis factor superfamily 8
vIPAG: Ventrolateral periaqueductal gray
WHO: World Health Organization
κOR: κ-opioid receptor
